# The Future of Cultural Psychology: An Interview With Jaan Valsiner

**DOI:** 10.5964/ejop.7575

**Published:** 2021-11-30

**Authors:** Jaan Valsiner, Carolin Demuth, Brady Wagoner, Bo Allesøe Christensen

**Affiliations:** 1 Department of Communication and Psychology, Aalborg University, Aalborg, Denmark

## Abstract

Jaan Valsiner (JV) has been the foremost cultural psychologist in the world for the last 30 years. In 2021 professor Valsiner turned seventy, and he agreed to do an interview with colleagues and students on his understanding of cultural psychology, its potential for innovation and its connection to his many interesting experiences from around the world. The interview was conducted by the three directors of the Center for Cultural Psychology in Aalborg Denmark: Carolin Demuth (CD), Brady Wagoner (BW), and Bo Allesøe Christensen (BA). For an extensive discussion of the different sides of Valsiner work, readers can consult the recently published Festschrift (Wagoner, B., Christensen, B., & Demuth, C. [Eds.]. [2021]. Culture as process: A tribute to Jaan Valsiner. Springer.).

**BW: To begin could you describe your own development in terms of cultural psychology? What was the situation when you were a student and started out as teacher/researcher and what has changed since then?**

**JV:** Well, I was very lucky because I lived in the Soviet Union and not only the Soviet Union but the peripheral area of the Soviet Union in Estonia. The Soviet Union was a deeply totalitarian country, as you know, but the periphery was ethnically different. Regarding Estonia versus Russia, Estonia was still under the political pressures but teaching was now done with Estonian language, and Estonians or Soviet Estonians were leaders in the Estonia. So, the education there was also limited on the one hand by a very strict curriculum coming from Moscow, somewhat modified locally. But at the same time carried out with people who are not very competent – or didn’t feel very competent in psychology. So, we as young students– I was the third wave of psychology students taken in - felt we were building something new in Estonia under the Soviet rule that was still highly influential.

The curriculum superimposed Russian psychology on us, and so we went to American psychology. The “we” here means more or less five or six people of the 25 taken in. The initial thing we started doing in the first year of the psychology study, was to do the impossible, namely translating the American personality research method (MMPI) into Estonian. The effort was very good for group formation and maintenance; we spent hours and hours discussing translations in a cellar of some dormitory. In the end we were broken apart, of course, and nothing came out of it but the important thing of this unity. We were doing something unified together and we were doing something new in this particular place.

Then after that I was lucky, indeed, to get some special program for studies which made it not mandatory for me to go to lectures – which I used completely. The last three years of my studies I never went to a single lecture. The professors were not happy and they punished me during the exams which I did have to take. So, I sometimes started to fail, sometimes quasi-failed and had to retake them, and then got poorly through. So it was clear that I was not a good student. But in these three years I was doing my own research step by step. That’s why you see me telling you that you do your own research and that’s the most important thing, because that is how you learn. Not by listening to lectures like mine and so on. Just do your research. You find out what you want to do, you find out what you DON’T want to do, which is equally important, and do something else. This kind of direct experience with research was very valuable at that time, and it got me a job at the university, first as a research assistant, then as an assistant professor.

[Contrib contnr1]As assistant professor, I also came into conflict with the university leadership: We were invited to send a review article to America to be published and suddenly the publisher required permission from the Soviet authorities, namely the university had to provide one little letter, saying that they have nothing against us sending this abroad. And I go to the vice rector and he says, “how old are you?”, “27”, “do you think you’re competent to write an article?” I say “yes”. “We at the university don’t trust you. The other vice rector, who is a chemist, is 52 and has never published internationally. How can you dare say that you are competent?” So out I go, no letter. Two weeks later the KGB interviews me in the secrecy of a personnel office. They have nothing to ask me, other than: “you are connected with the worst, do you know any anti-Soviet propaganda?” “no, no, no.” They even don’t want to recruit me but they have this meeting with me. Two years later I leave the country by coincidence. So that is in a way the starting point of my education. So you get education by doing, you resist the social system, even if it’s dangerous, because it was quite dangerous to insist on the request to publish something in the West, not physically dangerous but kind of professionally dangerous.

And then I actually became cultural psychologist 20 years later by coincidence. At first I considered it irrelevant but then gradually started to read more and more and think more and more of anthropological evidence and anthropological questions, and then sometime in the 1990’s the transition happened from development to cultural. So it’s a jump to 20 years later. Am I a cultural psychologist? I don’t know. When somebody asks me “are you a cultural psychologist?” I will answer with hesitancy, I don’t know. But also I don’t know if I’m a psychologist. I am a psychologist officially, but when I’m asked my identity I do not have it. Why don’t I have it? Because the responsibility of being a psychologist is probably too high, which I cannot take and carry.

**BW: Even though you may not identify as a psychologist, you definitely made important contributions to psychology. Where do you see your most important efforts?**

**JV:** Yeah, that’s a good question. I think I am unique in this cultural psychology theme by naively and adamantly insisting on *the irreversibility of time*. Naively in the sense of constantly emphasizing it. My friends are making fun of me “oh again he uses the word ‘irreversible time’”. Using it consistently it becomes almost trivial, almost common sense, that time is irreversible. So why do you need to emphasize it? Because it is not trivial.

I have two predecessors in doing that. Henri Bergson introduced focus on irreversibility of time in the 1890’s, in his philosophical take which I borrowed from, but I borrow it in the wrong way, not his way. Secondly Ilya Prigogine got the Nobel Prize in 1977, I believe, for bringing the notion of irreversibility of time into physical chemistry. So these are the two people who have used irreversible time very importantly and I think that their uses of them are only the beginning of the relevance of it in psychology. The implications of that notion are enormous: for example, it makes the notion of single case and single event central for our understanding of psychological phenomena. There is no repetition; there’s innovation over time but that is not the same. Something happens that is similar to the past but not the same, and it cannot be the same.

[Contrib contnr2][Contrib contnr3][Contrib contnr4]The second notion is my focus on *generalization*, and in recent years, the notion of what I call *hypergeneralization*, which is generalization beyond generalization. This is a difficult concept. I have difficulty explaining it myself at times but I think this is very central to understanding how human rationality is deeply affective. Affective worlds lead to new worlds of rationality, new versions of concepts exactly because there is this hypergeneralized feeling into the world. Usually you can talk about it in terms of values for instance. As an example, the whole mind becomes totally overwhelmed by some particular affective moment that is not reducible to a particular label. And I think that has interesting implications: one of them is that it actually allows us to transcend irreversible time, to encode particular values into the form of this nebulous hypergeneralized fields of meaning. When it feeds forward into the future it becomes kind of a catalyst in future relation to the concrete world. Take, for example, the notion of danger and the notion of safety. What we call ‘safety’ is a label but behind that is a huge hypergeneralized field. Now, we are taught safety by using our face masks, which is a beautiful example of a socially instilled illusion that cannot guarantee us anything. But we develop with this concrete task of wearing a mask; we develop this hypergeneralized feeling of being safe. And then it becomes translated into totally new contexts when safety becomes an issue which you cannot predict.

Similarly, you can never know at what moment the child will get into potential danger in a particular environment. The child has to act on the moment, and that moment of action, of avoiding the dangers, is exactly this hypergeneralized, internalized feeling of safety that suddenly becomes dominant. It is socialized but at the same time personalized. “I cannot do that; it’s a little dangerous. I don’t do that. I tell mother I didn’t do that because it felt dangerous” says the child. The mother says “fine, wonderful”. In contrast, those not having this kind of a background of nebulous orientation simply do anything and they end up in danger and in actual accidents. That’s the second contribution, I don’t think there are any others.

All of this comes from the focus on semiotic dynamics where my unique perspective involves signs, operating upon signs, operating upon signs, etc. So this is a semiotic dynamic notion but this is secondary to the two very general issues.

**CD: That leads me to another question: how does the irreversibility of time lead into methodology? You already mentioned there is a danger of putting things into method boxes. Recently, you argued that if cultural psychology will have any future it is determined by whether we will have innovative methodologies. This related to irreversibility of time and to the nature of open systems. Can you elaborate a bit on what would be the methodological approaches here?**

**JV:** Most of our methods in psychology are flat. Flat means what? I as an interviewer ask you a question. You as our interviewee, you give me an answer. I as the researcher go and interpret your answer at the manifest level. I collect the answers and I now have the data. This is flat. It can be an interview, it can be a questionnaire, or it can be anything really. All the testing is flat because it creates basically commonsense answers to commonsense questions. Sometimes these are very complicated questions if you look at personality questions and items.

Now escalating questions are the ones that explicitly trigger something in the person. In the experimental context, you will do something nice or nasty to people. Americans in the 1920’s in order to elicit emotions would show their subjects dead rats in the laboratory to elicit emotion. It would be crazy but nevertheless very American.

Now, what is “deep”? The idea comes to me from listening to the music of Arvo Pärt. Have you listened to music of this Estonian compatriot of mine? Try, for example, to listen to the small piece for Alina. It’s a very slow music, where the focus explicitly is on the sounds that are disappearing. That’s why it has to be very slow. Instead of dun-dun-dun-dun it’s like duuun… duuuuun. And this is exactly where the issue is, when you think of human beings, not as immediate reactors but co-constructors of particular meaning. You want not to have the first answer that the person gives you, give a question today, answer today. You want to find out a week later, if the person has thought about that question. You do an interview today, a week later you call the person and say “do you remember what happened in our interview? Have you been thinking of any of it?” and out of the ten topics maybe about two of them the person will say, “every day I think about it”. This is the deep method, because you now know from the memory trace or the memory confession that the particular item you brought into the person’s domain, has been reverberating, so to say.

It’s very similar actually to Bartlett’s (1932; see also Wagoner, 2017) repeated reproduction method that Brady has been using (e.g., Wagoner et al., 2019; Wagoner & Gillespie, 2014), only it’s a little bit different, because the process of actually remembering of the next retelling or their process of not losing or confabulating something is actually part of the continuity, so to say. So that would be an example of the deep method, because the only thing you add is a kind of follow up using memory trace to look at their construction of the actual answers, the meaning-construction in the personal world. Very simple: it can be done with interview, it can be done with specific questionnaires even, and it can be done online.

**CD: And you said you got the idea about the deep methodologies from listening to music and I know you also have a general interest in art. At one point recently you also said that analyzing verbal data is not able to capture affective-field-like signs. So I’m wondering to what extent do you think art can help us, to study art or to use art in our cultural psychology research?**

**JV:** That’s what I’m now doing since I’m very lazy: all my subjects are 400 years old! They do not need permission from the human ethics committee to be studied. They do not need to fill out signed consent forms. That’s why the study is very free, but that is only a joke, not more than that.

More importantly, what art would tell us is not just art itself, but the whole specific art emergence in a particular societal world. That’s why, for example, at this moment, one of the new projects I am involved with is I am looking at the emergence of a psychological understanding in Rudolph II’s time, the Holy Roman empire. That is exactly 1572 to 1612, a very strange time for a psychologist to look at. However, as it is exactly at that time that psychology starts as a new discipline—Not 1879, not 1806, but around 1580’s, 1590’s. In the baroque atmosphere of the Rudolph times, there’s a very interesting confluence between astronomy, Johannes Kepler, alchemy, astrology of whom Johannes Kepler was one actually, for the parallel between astronomy and astrology for Rudolph II, and art. So the interesting issue is to see how the specific art was utilized or produced in that specific time in Prague, which was an open time for arts and sciences and also relatively free from political pressures which came later with the Thirty Years’ War.

So how does all of this lead to specific ideas? I don’t know what comes out of it, but it is one of the ways of trying to look at *allegories*. The notion of allegories, all over the place in art, is one the challenging topics for me right here: What made something into an allegory? How can we understand an allegory? All of the 16th century discourse was basically allegorical. They were verbally or nonverbally allegorical about something else, and this something else is a very general, hypergeneralized kind of knowledge which is difficult to trace. So I’m getting myself into very difficult domains in this case.

Of course, more ordinary ways of looking at art are very simple. We use art all the time, producing it, asking our subjects to draw and paint, presenting it, asking ourselves, interpreting it. This is all known in psychology; nothing new here. But in terms of contrast with the verbal channel, it allows us some access—but it is difficult to interpret this access—to what I call ‘up-conscious’ and also Freud’s unconscious, maybe. These are temporarily separated from the verbal domain. The same with music: When you look at musicians they will be very explicit that you cannot put into word what is in music. And music was, after all, historically a very central phenomenological starting point for most of the turn of 20th century psychology, and late 19th century psychology.

This leads us to a question of the role of history, and interesting enough the role of art, art history and psychology, so to say. What people do before they become psychologists, of course, has big relevance to how they think as psychologists. So if you are a 19th century young person coming out of very often theologically minded households, classical music is something that you learn to play, listen to, and sometimes compose. Therefore, music is everywhere in their efforts to understand psychological phenomena. Sometime in the early 20th century, this musical focus disappears from psychology and is replaced by a focus on visual perception as the primary field. So, the psychologist’s history in music, history in phenomena (i.e. societally relevant ones), came from a particular impetus and then started to disappear.

**BA: I would like to ask something about your experiences in Estonia, because I was thinking about the critical role of the psychologist or cultural psychology today. It seems to me like what you’re describing is, on the one hand, two parts of the notion of critique: in one sense it’s pointed towards opening up the boxes, thinking about the conditions of possibility. And in the other sense, it’s kind of like a practical role of the cultural psychologists as well. You sometimes describe it as “going against the inertia of the systems”, or the “catch 22s of the systems” as well. So I was thinking about whether you could say a little bit more about whether there is a need for a more theoretical and practical role for cultural psychology as a critique today?**

**JV:** Well, the practical role is absolutely essential for each and every one of you, especially in your studies of the cultural psychology because you keep asking the question “What can we do with it?” and we are not very good at answering that question. We are trying our best to do it, and the usual answer is that it gives a perspective that allows for translation into very concrete social life contexts. But what they are is almost the same as telling the child “be careful in the future”. But now, to be more concrete, this links back to the need for sociology and psychology, basically.

In order to understand why a cultural psychology can do something, it’s probably important to start from finding out what it cannot do. In another sense, the first diagnosis of the social role of a psychologist, and this may mean decision, which job you want to take as a psychologist looking for a job after your candidate. Looking for one month, two months, five months, ten months and so on, and rejecting the first five because they do not fit you. It is exactly here this diagnosis becomes very important “can I do the work this job insists on?” “am I happy with it?” “Is it exactly what I want to do after my cultural psychology build up in these two years?” and if the answer is no, you just don’t take the job. If the answer is “I can do it”, you try, how long you last is another question.

In another sense, your own build-up of the specific understanding of being a person in a given society, e.g. the Danish society, would lead you to see where your expertise in cultural psychology is applicable. What kind of expertise is this? Quite obviously it’s an expertise in meaning construction. Not only by you but by another person. As specific adjustment, as help in the meaning construction to the person if the person is ready for it, if the person is not resisting, neutralizing and so on. This is another aspect of your preparation as cultural psychologists: are we good at preparing you, I don’t know, I think not. You will know it better than I can say but, at least this is something to be discussed, something to be very explicitly put on the table so to say in the Masters program, saying “we want that as cultural psychologists after our masters”. We can do it, but it depends on individual interest, individual orientation, where you want to go after your Masters, what kind of positions you are looking for, and this is all individually specific. No doubt it’s necessary because that depends basically on the longevity of this program and any other cultural psychology program. Whenever I hear that a new cultural psychology Masters program has opened, I always ask “what else what else is linked with it? Clinical, organizational, something else? So perhaps we should start to specify what kind of cultural psychology that could be, because it’s not something ready for practical use in society, and fortunately so because that means you’re less boxed in.

You mention critique. What I would emphasize very strongly and also you see me dismissing some areas of psychology through analysis of their basic assumptions. This is actually a very constructive critique; you dismiss many areas exactly because you concentrate on a clearer view of others. This may actually apply also to something you need to go beyond boxes in the reading of literature. Nowadays, with the enormous proliferation of published work in psychology, to which we ourselves contribute, what becomes necessary is not so much what to read but how to read. You need to learn to scan existing literature very quickly and very quickly decide “I don’t need it”, to concentrate on those very few pieces that you actually need.

**BA: I wanted to ask about the notion of facts and more broadly facticity. You sometimes use the word “Gegenstand,” implying resistance from the world. So, do we need some notion of fact beyond a behavioristic, positivistic way of thinking about it?**

**JV:** Yeah, that is why I’m starting to operate with the notion of *Gegenstand*. And you’re right, it’s probably in my terms it would be the equivalent of facts getting away from behavioral fact, because behavior is not a fact. Behavior is something observable okay. The G*egenstand* notion gives us a minimalistic structure of a movement tendency towards an obstacle and the resistance to it. So the notions of movement towards, resistance to and the barrier or border is quite important.

This brings me to another question you have listed here which is a question of ethnomethodology. Because ethnomethodology, if my understanding is correct, is a very clever method of crossing the border, the Gegenstand border, or testing the Gegenstand border. Garfinkel (1967) sent his students back home for holidays and told them to operate as if they are lodgers in their own homes. So they will have to ask the mother for permission to open the refrigerator “may I take some drink from the refrigerator?” driving the parents crazy: “for 16 years you never ask for my permission, why are you doing it now?” But notice what is happening, it’s exactly that the manipulation is the moving of the border, suddenly introducing a border; I cannot open the refrigerator any more, I have to ask permission and the particular resistance to it from the parents is basically a specific aspect of dealing with it, so on that point of view the ethnomethodology of some sort would theoretically fit very interestingly into the whole scheme. The natural nature of this experiment-- the mother doesn’t know what the professor has asked the son or daughter to do-- is in a way cheating, but on the other hand it’s a very informative cheat.

**BW: I’d like to ask more about experiments. They’re often considered the royal road to knowledge in psychology. Where do you see their possibilities and limitations?**

**JV:** Well, an experiment is a structure or a modification of a whole structure of the field, in a gestalt sense. And this whole structure of the field in terms of cultural psychology, of course, is a structure of the meaning of the field. The meaning of the field is set up by the person on the basis of some experimenter marking the particular field in particular ways. So we are now involved in a remarkable experiment of this kind, which is that of interpersonal distancing. Our social orders give us a demand to be distant until the virus carries over, at least. But the interesting aspect is how that starts to change all of our particular ways of feeling, not just acting, but feeling. And with that, we are basically looking at specific outcomes of this. So we have the modification of the total field structure together with a modification of the feeling into the particular. Now at least a very interesting resistance, which you already see happening in the Gegenstand structure. New barriers are introduced: from Thursday we will all be in masks here, and the resistance to that is going. The resistance is not to the virus, the resistance is the social politics involved, now over the virus, we are even almost over the fear of economic collapse, now we are entering the phase of political, socio-political conflict. We can present it as a Drama of the social power of the institutions and the personal feelings of freedom. This is a very interesting period, because you see already in different countries, direct protests about all kinds of restrictions, you see those kidnapped by different political forces for that purpose, so this is an opportune moment to look at the emergence of societal discontent, at the societal level, which is parallel to the emergence of personal will to act against the system of rules. This need not happen in the domain of rules introduced by the virus or for the virus. It can happen with something else; it can be displaced from this domain of knowledge to another domain. It can be an elaboration of racial discourse; it can be an elaboration of refugee crisis in some different forms, new forms. I don’t know. But the idea is that the specific resistances to societal interventions are likely to lead to some new forms of action. But it’s not an answer to your question, it’s kind of an elaboration of it, I probably cannot answer your question.

**BA: these kind of actions can be both positive and negative?**

**JV:** Sure, absolutely

**BA: So wouldn’t the cultural psychological critique have some sort of obligations to watch society at large, to have an agenda regarding stuff like this?**

**JV:** Yes, of course, absolutely

**BA: you said before that the thing about diagnosis was it’s kind of like a tool. So I want to ask you about technology. I think that’s a key cultural psychological factor because technology is never neutral, it’s always mediating people’s relationships, it could be interesting for us to hear a bit about what you think about technology, how one could use that in a cultural context?**

**JV:** Well, first of all, it is, of course, a perfect example of cultural construction. It’s a very objective outcome of cultural construction; nobody can invent the cellular phone without having very specific meaning systems already encoded into the making of it. Once the cellular phone is invented and once it proliferates everywhere in the world, it radically changes all of our ways of handling memory. For example, memory will die out as a result of cellular phones, as long as there’s electricity, of course, if we lose the battery, suddenly we are in deep trouble very soon because there is no alternative anymore. So, in this sense technology is very central. Both how new technology produces and new conditional meaning construction introduces new meanings and leads to more meanings, and the dynamics of the particular transformation is describable in a relatively short time, which is a value. It would also be interesting to link it with history: we are now talking about cellphones and their specific roles in our self-construction, but what about previous technology, what about the Luddite movement in the 19th century, of violating or breaking machinery in factories, basically an act of preventing technology from developing. What about our present day paranoid discussions about China trying to interpenetrate by Huawei all of your personal secrets. The very idea of these kinds of discourses is a conspiracy about China that every cellphone will be immediately connectable with the Chinese government. So all of this is very interesting additional cultural psychological phenomena not yet studied, and they will have enormous societal benefits too, if you prove conclusively that most of the fears of the espionage with cellphones are either simply impossible or possible but at the same time unrealistic. For example, back in the Soviet Union, we always felt that every letter we sent abroad would be read by the KGB. This was not possible; they didn’t have enough labor force to do it, so they were selective, of course. Now we are not worried at all about Google registering each and every move we make almost anywhere. We are not paranoid about Google’s interference, why? Why do we trust Google and their helpful suggestions that you must buy this and this and this, in contrast to Huawei. This occurs not only at a societal level but also at a personal level; we are worried about the virus but we are not worried about our own cellphones, which is interesting. In fact, Google gets everything from us.

**BA: Do you think there’s a difference between the “IT generation” which are “born” into an online context and us who are a little bit older who are familiar with an offline/online distinction?**

**JV:** Oh of course, there has to be, because meaning construction in the new generation starts from a different baseline already. That’s what the child already gets very early, what we could only wish to have in our adolescence. Every next generation will innovate. The question is what are the specific meanings, in which direction does innovation go? And again let me come back to the consumer notion that I mentioned before. Every generation has new technology which makes it remarkably easy to make choices in that dating app: you’ll make lifelong choices dating, left, right, left, right, left, right. Versus developing a particular elaborated construction of something. You build your home on your computer first and then you build the real home, for example. The constructivity versus choice distinction becomes very central. It can go both ways. You can build new technologies that demand a high construction orientation, or you can build new technologies that demand just a consumer orientation. This distinction also goes beyond technology; this is a societal agenda beyond technology. You use technology to study the societal agenda, in some sense.

**BA: some people have been talking about “Web 3.0” where we see a change. Where we usually try to make technology adjust to us. A lot of practices actually aim at adjusting us to this technology, and with these practices created by big tech companies.**

**JV:** Well, you see, there is exactly the research project; “how do people make sense of this kind of loss of freedom?” basically it’s a loss of freedom, but it need not be presented as a loss of freedom. It can also be presented as opportunity.

**BA: Some of them are not aware that they are not free, but after they have been told that they are not free, and they don’t mind. They want to be kept in capture.**

**JV:** Well, that may be a societal principle we know from very ancient times, maybe.

There was some other interesting question here… “learning from and not just of the world”.

I think this is also the fact one. I will say that we learn not only from, but with others, not about the world.

**CD: maybe we can take a final webinar question? Imagination also plays a big role in your work, and you said you have looked through the last 25 years of cultural psychology to see where we have been going. Now if you try to imagine the next 25 years of cultural psychology, where do you see current developments that you find are very fruitful and promising to follow?**

**JV:** I see, actually, quite a few, very young, very often student level psychologists developing very sophisticated new ideas and that give very much promise for the future. The irony is that it’s exactly not only the young who make innovations, but they are also the ones who have the opportunity to do it, because at a certain moment, the opportunity becomes less. So I’m actually quite positive about the future, despite of my concerns, but I am not positive on the institutional sense but on the human sense. People taking active interest, discussing their ideas, especially worldwide. You see very strong development in South America, you see it in Asia, it’s a very strong worldwide movement this moment, and something of there will develop something new.

**BW: Any final questions, or would you like to say a concluding word?**

**JV:** Thank you for all the questions, thank you for the discussion, I’ve learned quite a bit from you today. So thank you.
